# Long-Term Stability of the SGA-WZ Strapdown Airborne Gravimeter

**DOI:** 10.3390/s120811091

**Published:** 2012-08-09

**Authors:** Shaokun Cai, Kaidong Zhang, Meiping Wu, Yangming Huang

**Affiliations:** College of Mechatronics and Automation, National University of Defense Technology, Changsha 410072, China; E-Mails: kdzhang@263.net (K.Z.); meipingwu@263.net (M.W.); huangyangming@gmail.com (Y.H.)

**Keywords:** strapdown airborne gravimeter, long-term stability, drift, quadratic curve

## Abstract

Accelerometers are one of the most important sensors in a strapdown airborne gravimeter. The accelerometer's drift determines the long-term accuracy of the strapdown inertial navigation system (SINS), which is the primary and most critical component of the strapdown airborne gravimeter. A long-term stability test lasting 104 days was conducted to determine the characteristics of the strapdown airborne gravimeter's long-term drift. This stability test was based on the first set of strapdown airborne gravimeters built in China, the SGA-WZ. The test results reveal a quadratic drift in the strapdown airborne gravimeter data. A drift model was developed using the static data in the two end sections, and then this model was used to correct the test data. After compensating for the drift, the drift effect improved from 70 mGal to 3.46 mGal with a standard deviation of 0.63 mGal. The quadratic curve better reflects the drift's real characteristics. In comparison with other methodologies, modelling the drift as a quadratic curve was shown to be more appropriate. Furthermore, this method allows the drift to be adjusted throughout the course of the entire campaign.

## 1. Introduction

The measurement of the Earth's gravity field is quite important in many fields, including geophysics, geodesy, and geodynamics. Airborne gravimetry is a method of determining information about the Earth's gravity using an aircraft-borne gravimeter. This method can provide gravity information in terrains of difficult access and in areas that contain both land and ocean [1].

Different airborne gravimetry systems use different principles, such as the 2-axis stable platform (e.g., LCR Air/Sea Gravity System), the gimballed inertial navigation system (e.g., GT-1A, AIRGrav), and SINS (e.g., SISG, SAGS, SGA-WZ) [2].

Airborne gravimeters using SINS have significant advantages. These gravimeters are small, light-weight, and cheap, with low power consumption and a simple structure. In addition, they have a high data output rate for detailed post-mission analysis, are easy to operate during flight, and can implement vector gravimetry. The first study of the strapdown gravimeter system was performed by Schwarz's group, whose SISG strapdown system has an accuracy of 1.5 mGal and a resolution exceeding 5 km. The Honeywell LASEREF III SINS used by this system is a commercial product and struggles with some hardware transformations, such as thermal control. In general, it is difficult to use this system in engineering applications; there has been no further development of this system after 2001 [3,4]. The Institute für Erdmessung und Navigation of the University of the Federal Armed Forces in Munich (Germany) designed a strapdown system using the SAGEM Sigma 30 INS. However, public reports about this system's accuracy are lacking [5,6]. The Bayerische Kommission für die Internationale Erdmessung of the Bavarian Academy of Sciences and Humanities also developed a strapdown system named SAGS. This system consisted of three fiber-optic gyroscopes and four high-performance QA3000 (30) quartz flexible accelerometers. However, this system's thermal control accuracy cannot meet the requirement and practical results have yet to be achieved [7,8]. In contrast, the self-designed SINS used in our system can be further tailored to accommodate the specific needs of airborne gravimetry, such as thermal control and shock absorption. The accuracy of our system is comparable with that of GT-1A.

The accuracy of the SINS is dominated by the long-term drift. Because SINS is a primary, critical component of the strapdown airborne gravimeter, the accuracy of a SINS-based strapdown airborne gravimeter is also influenced by the long-term drift. After installation in the aircraft and the initiation of the airborne gravimetry measurement, the strapdown airborne gravimetry system cannot be turned off until the campaign is completed. However, the campaign often lasts a very long time, sometimes over a month, which exacerbates the negative effects of the long-term drift.

To address this problem, some researchers have proposed a method of level adjustment in which a constant is used to improve the data accuracy [9]. However, this constant-value model can only compensate for bias, not drift. Some researchers compensate for the drift using end-point matching [10–12], which models the drift as linear and uses the referenced gravity at the two ends of the survey lines. This linear model is only applicable for short flight times due to the non-linearity of the actual drift. Using this model, we must adjust the drift of the gravimeter each day. Some researchers also model the drift as linearity and use the crossover adjustment to estimate the drift's parameters [13,14]. In reality, the crossover method with a linear model, which is often used in gravimetry, is a piecewise linear method. If the grid survey lines cannot be obtained, the crossover method cannot be used. Moreover, the crossover method can be used to adjust the drift but not the bias. In some papers, the drift is modelled as a polynomial [15,16], which is another case for which the crossover method is necessary. To adequately characterise and compensate for the drift, a long-term stability test was performed using the first strapdown airborne gravimeter developed in China, the SGA-WZ.

## 2. System Description

**Figure 1.**
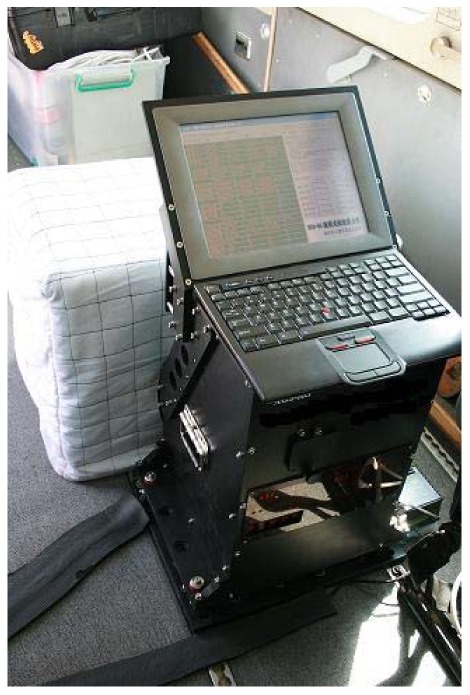
Photograph of the system.

The strapdown airborne gravimeter mentioned in this paper was developed by the Laboratory of Inertial Technology of the National University of Defense Technology. This system consists of a high-performance SINS, a Global Positioning System (GPS) receiver, an anti-vibration system, a data logger and post-processing software. The flight test using this system was performed in Shandong Province of China with a flight altitude of 400 m and an average airplane velocity of 60 m/s. The test results show that the system repeatability is 1.5 mGal for a spatial resolution of 4.8 km [17]. The major advantage of this system is reliability. A photograph of the system is shown in Figure 1.

Component 1: SINS is mainly used to measure the aircraft's specific force and attitude. The accelerometers used in this system are three quartz flexible accelerometers with a stability of ±0.2 mGal and a scale factor uncertainty of ±3 ppm. The gyroscopes are three ring laser gyros with a stability of ±0.004°/h and a random noise of ±0.002°/√h. The accelerometer installed in the vertical direction was also used as gravity sensor. SINS has two main functions: measuring the specific force, which contains the gravity information, and measuring the aircraft's attitude.
Component 2: The main function of the GPS is to obtain the aircraft's position, velocity, and acceleration. For that purpose the relative GPS positioning mode is used therefore, besides the receiver installed in the aircraft, GPS master stations on the ground are needed.
Component 3: The anti-vibration system can isolate and thus reduce the effect of the high-frequency vibration from the aircraft's engine.
Component 4: The data logger is used to collect INS and GPS data, monitor the state of the entire system and control the process.
Component 5: Post-processing software is used to calculate the gravity disturbance from the raw measurement.

In addition, the thermal control is used to provide a stable working environment for the gravimeter, with an accuracy of 0.02 °C. Before temperature stabilization, the system must be preheated for ten hours. If the system is turned off on one day, it must be preheated for ten hours the next day. Therefore, the system cannot be turned off during the campaign.

## 3. Test Description and Data Processing

The gravimeter was placed on a marble platform, and static data were recorded. Because of the inertial sensors, the system must be preheated after being turned on to work normally. The system was kept working for 104 days from 25 February 2011 to 8 June 2011. During testing, the state of the system was checked three times a day. These checks indicated that the system was free of errors for the duration of the test, indicating good reliability.

The data obtained from this test contain some high frequency noise, which can be filtered using a low-pass filter. The observed data from the gravimeter should be processed and analyzed using the following procedure.

**Figure 2.**
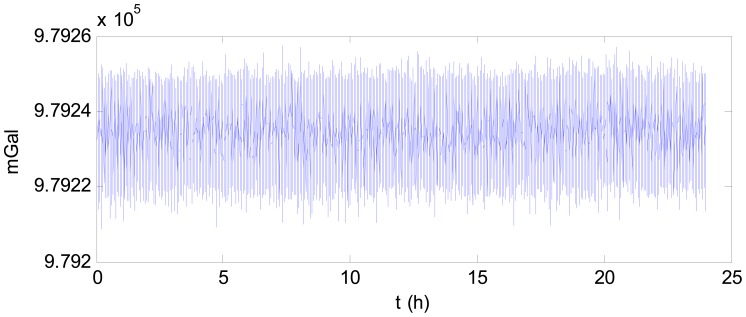
The raw gravimeter data on 27 May.

**Figure 3.**
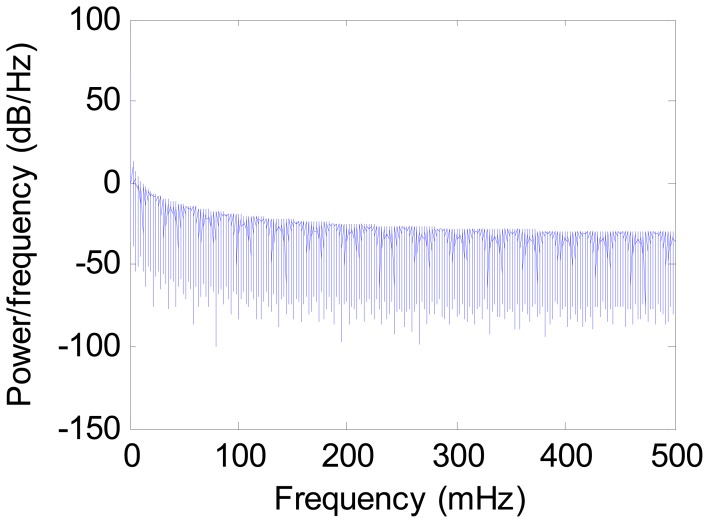
The power spectral density of the gravimeter data before filtering (27 May).

**Figure 4.**
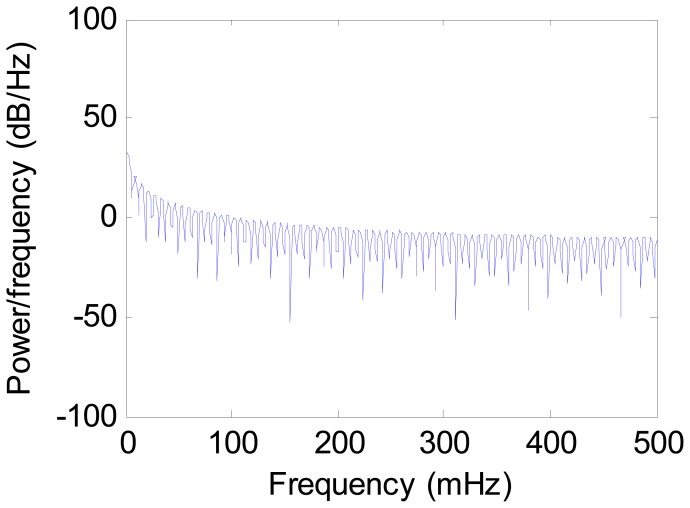
The power spectral density of the gravimeter data after filtering (27 May).

**Figure 5.**
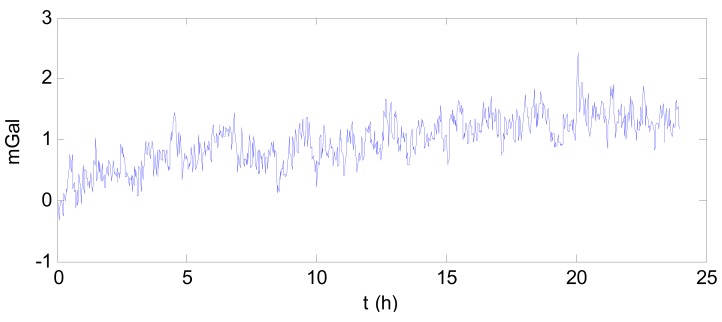
The observed data on 27 May (after filtering with the starting value subtracted).

Step 1: Filter the gravimeter data using a low-pass filter to remove the high-frequency noise. In this test, the simple segmented averaging was adopted. This simple low-pass filter can help to reduce the data collected from the long time testing. Take the data collected on 27 May for example. The raw gravimeter data is shown in Figure 2. The power spectral density of this data is shown in Figure 3.
  The gravity is the low frequency information which occupies very narrow range of frequency near zero. As Figure 3 shows, there is high frequency noise in the observed data. After filtered by low-pass filter, the power spectral density of the data is shown in Figure 4.
Step 2: Subtract the value measured at the starting time from the subsequent data to facilitate analysis and observation of the drift. Figure 5 shows the observed data collected on 27 May, which was subtracted by the value measured at the starting time after filtered.
Step 3: Perform statistical analysis of the resultant data obtained from the above two steps.

## 4. Results and Discussion

**Figure 6.**
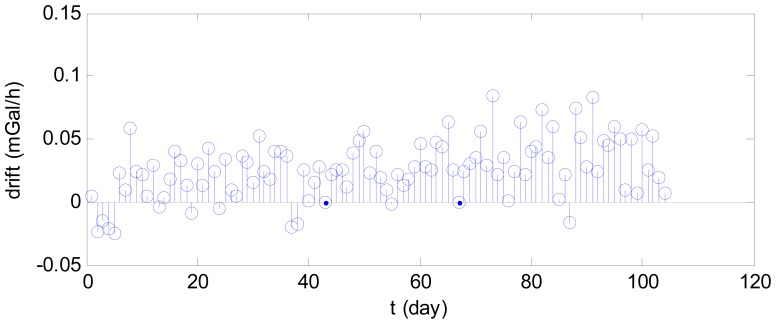
Drift in gravimetric data with time.

The drift of the post-processing data was presented in Figure 6, which shows that the mean daily drift generally increased with time.

**Figure 7.**
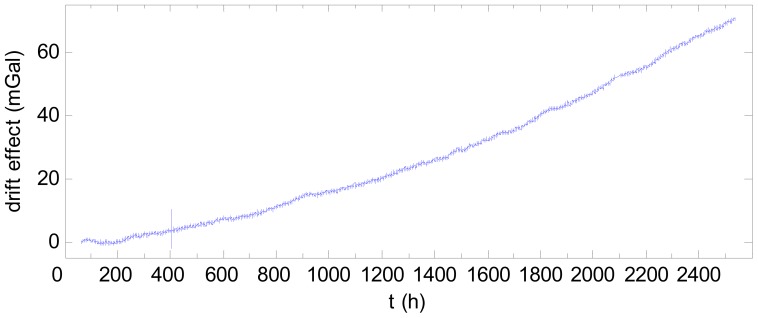
Gravimetric data after filtered and subtracted by starting value.

Figure 7 shows the 104-day data after filtering and subtracted by the starting value. A drift is clearly visible, with the drift effect of the final day being 70 mGal greater than that of the first day. This drift negatively impacts the accuracy of the gravity measurement. In Figure 7, there is an abnormality near 400 h, (*i.e.*, 2 pm on 11 March 2011), which may be attributed to the major earthquake occurring at the time off the northeast coast of Japan.

**Figure 8.**
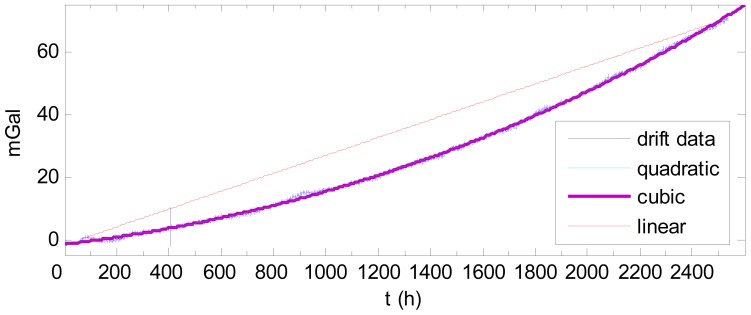
Curve fitting.

To illustrate the results obtained using the traditional method, the drift shown in Figure 7 is fitted by a linear curve using the data at the two end-points. The drift is then fitted by quadratic and cubic curves. Comparing the fitting results, shown in Figure 8, the quadratic and cubic curves produce good fits, but the linear curve does not describe the drift well. The statistical results of the differences between the fitting curves and the drift are shown in Table 1. The statistical analyses do not account for the abnormality caused by the aforementioned Japanese earthquake.

As Table 1 shows, the quadratic and cubic curves are superior to the linear curve. The linear curve can produce an error of as much as 13.61 mGal, whereas the quadratic and cubic curves fit the drift much better, with errors of less than 2.5 mGal. The quadratic curve and cubic curve fit the drift in the same accuracy; thus, cubic fitting is not necessary.

## 5. Application of the Drift Compensation

Airborne gravimetry campaigns always involves dynamic measurements. However, static data can be used to adjust the gravimeter's drift and bias. Static tests are generally performed before the aircraft takes off and after it has landed. These static tests are conducted at the same location. Static data are necessary to fit and compensate for the gravimeter's drift; additionally, larger static datasets produce more accurate drift models. Therefore, longer gravimetry campaigns should include longer static tests.

**Figure 9.**
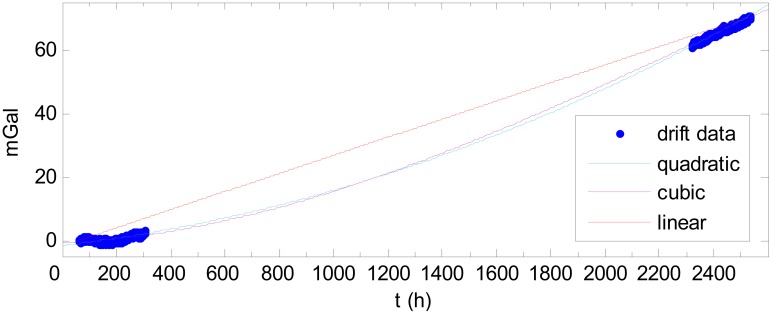
Curve fitting using the static data.

**Figure 10.**
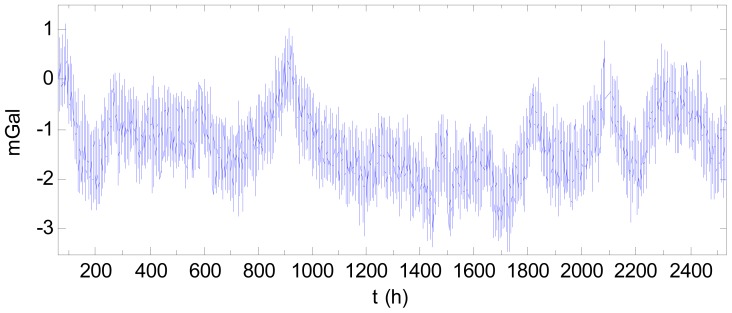
Result after the drift was compensated using a quadratic curve (after subtracting the starting value).

**Figure 11.**
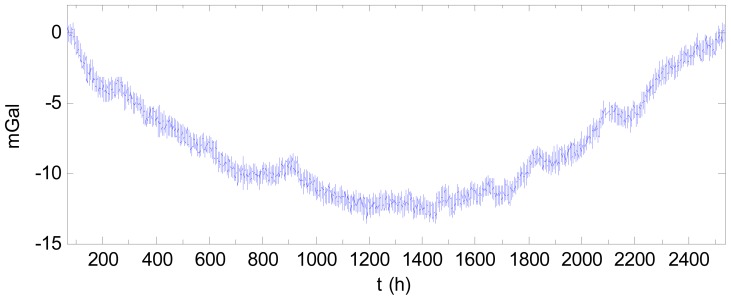
Result after the drift was compensated using a linear curve (after subtracting the starting value).

**Figure 12.**
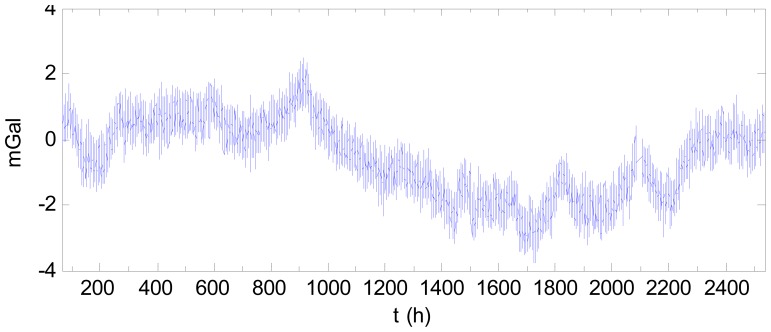
Result after the drift was compensated using a cubic curve (after subtracting the starting value).

As an example, consider the data mentioned above. Fitting the drift with a quadratic curve using the data for the first 10 days and last 10 days produces the result shown in Figure 9. The compensated result using quadratic curve is shown in Figure 10 after subtracting the starting value. Comparing Figure 10 with Figure 7 indicates the drift was compensated well. In Figure 10, the drift effect with regards to the starting time is only 3.46 mGal, which is much less than the pre-correction value of 70 mGal. For comparison, the results using a linear fit based on the two end-points and using a cubic fit of the data for the first 10 days and last 10 days are also shown in Figure 9. The compensated results by the linear and cubic models are shown in Figure 11 and Figure 12, respectively. The statistical results of the differences between the fitting curves and the drift are shown in Table 2. As Table 2 shows, when fitting the drift using static data in the two end sections, the quadratic fitting remains the best and the linear fitting is still poor while the cubic fitting worsened. Thus, modelling the drift as a quadratic curve is beneficial in practice.

## 6. Conclusions

This study has shown that the drift of a strapdown gravimeter has a quadratic behavior. After adjusting the drift using a quadratic curve produced by the static data in the two end sections, the maximal drift effect decreased from 70 mGal to 3.46 mGal. Compared with other methods, quadratic modelling best reflects the drift's real characteristics and allows the drift in the entire campaign to be compensated simultaneously. However, more dynamic tests should be performed to verify this method's reliability. Furthermore, the following considerations should be taken into account:

1. In airborne gravimetry campaigns, static data collected during the campaign when the aircraft is on the parking apron can be used in addition that from the campaign's beginning and end. The use of more static data provides a more accurate model.
2. When the referenced gravity at the parking apron is known, it should be used to implement a constant correction as well.

## Figures and Tables

**Table 1.** Fitting results statistics (mGal).

**Fitting**	**Maximal Difference**	**Fitting Accuracy**
linear	13.61	8.86
quadratic	2.48	0.57
cubic	2.36	0.56

**Table 2.** Statistics of the compensated results (mGal).

**Fitting**	**Maximal Difference**	**Standard Deviation**
linear	13.61	3.66
quadratic	3.46	0.63
cubic	3.80	1.15
